# Renal Infarction in SARS-CoV-2 Infection: A Case Report

**DOI:** 10.7759/cureus.62415

**Published:** 2024-06-15

**Authors:** Mohamed A Baghi, Nishan K Purayil, Vamanjore A Naushad, Irfan Varikkodan, Khaled Mohamed S Alarbi, Elmukhtar Habas

**Affiliations:** 1 General Internal Medicine, Hamad Medical Corporation, Doha, QAT; 2 Internal Medicine, College of Medicine-Qatar University (QU) Health, Qatar University, Doha, QAT; 3 Clinical Medicine, Weill Cornell Medicine - Qatar, Doha, QAT

**Keywords:** covid-19, arterial thrombus, kidney infarction, acute kidney injury (aki), anticoagulation, coronavirus disease 2019 (covid-19)

## Abstract

The novel coronavirus disease 2019 (COVID-19) is an infectious disease caused by SARS-CoV-2 and associated with a wide spectrum of clinical manifestations ranging from asymptomatic carrier states to fulminant respiratory distress and multiple organ dysfunction. The intravascular arterial and venous thrombotic phenomena are one of the most prevalent and devastating consequences and tend to occur in patients with a severe disease state. Here we present a 45-year-old male with a medical history of essential hypertension (HTN) who presented with severe left flank pain accompanied by dry cough and fever for five days. He was found to have acute kidney injury (AKI) with concomitant renal infarction in computed tomography angiography (CTA) in the setting of a COVID-19 infection. He was eventually managed with novel oral anticoagulation (NOAC) and was discharged after a short hospital stay. Follow-up thereafter showed stable baseline renal function with no relevant symptoms.

## Introduction

The hypercoagulable state related to SARS-CoV-2 has been significantly linked to macrovascular and microvascular thrombotic phenomena and is thought to be the most important predictor of unfavorable outcomes, such as hospitalization, admission to the intensive care unit (ICU), and death [1]. Several studies have linked severe COVID-19 infection with venous thromboembolic phenomena, including but not limited to pulmonary embolism, myocardial infarction, stroke, and aortic thrombosis [2]. However, the underlying mechanisms are not fully understood and are attributed to multiple complex interactions of direct vascular endothelial cell injury, excessive pro-inflammatory cytokine release, sustained activation of the complement system, and systemic fibrinolysis shutdown [2, 3]. This report illustrates a unique case of renal infarction in the setting of a COVID-19 infection in a fully vaccinated patient.

## Case presentation

A 45-year-old male presented to the emergency department (ED) with a four-day history of severe left loin pain associated with fever, dry cough, and mild throat pain. He did not report any shortness of breath, hemoptysis, weight loss, or urinary symptoms. His medical history was significant for essential hypertension, and he was on telmisartan and aspirin for primary prevention. There was no family history of thromboembolic or rheumatological illnesses. He was an active smoker and had received three doses of the Pfizer vaccine; the last dose was a year ago. At presentation, his initial vital signs were: temperature: 37.5°C, blood pressure: 120/76 mmHg, pulse rate: 71/min, respiratory rate: 19/min, and oxygen saturation: 100% on room air. A physical examination showed a normal oropharynx appearance with significant left-costovertebral angle tenderness. The initial laboratory investigations revealed leukocytosis of 14,000 leukocytes/µl, elevated C-reactive protein of 144 mg/l (normal range <5 mg/l), creatinine of 133 umul/L (normal range 62-106 umul/L), and minimal alteration of hepatic transaminases (aspartate transaminase (AST): 49 U/L, alanine transaminase (ALT): 103 U/L). The urine dipstick analysis showed no evidence of microscopic or macroscopic hematuria, and the nasopharyngeal swab for SARS-CoV-2 RNA polymerase chain reaction (PCR) was positive (Table 1).

**Table 1:** Result of laboratory investigations during the patient's hospitalization and follow-up HDL: high-density lipoprotein; LDL: low-density lipoprotein; INR: international normalized ratio; APTT: activated partial thromboplastin time; ANA CTD: anti-nuclear antibody connective tissue diseases; ANCA: antineutrophil cytoplasmic antibodies

Laboratory variables	First day of admission	Second day	Fourth day: discharge	At the four-month follow-up	Reference range
White blood cell count (x103/uL)	14	7.8	7.7	6.8	4 - 10
Hemoglobin (gm/dl)	12.3	11.8	11.9	13.8	13.0 - 17.0
Red blood cell count (x106/uL)	4.4	4.4	4.1	5	4.5 - 5.5
Hematocrit %	38.7	36.9	36.9	43.0	40 - 50
Platelet count (x103 /uL)	254	250	260	253	150 - 400
C-reactive protein (mg/L)	144	-	134	-	0 - 5
Procalcitonin (ng/mL)	0.2	-	-	-	<0.5
Alanine aminotransferase (U/L)	103	-	47	-	0 - 41
Aspartate aminotransferase (U/L)	49	-	12	-	0 - 40
Bilirubin T (umol/L)	8	-	6	-	0 - 21
Alkaline phosphatase (U/L)	73	-	82	-	40 - 129
Creatinine (umol/L)	133	103	95	92	62 - 106
Urea (mmol/L)	5.3	2.8	4.4	5.2	2.8 - 8.1
Sodium (mmol/L)	139	138	140	141	135 - 145
Potassium (mmol/L)	3.8	4.2	4.1	4.6	3.5 - 5.1
Chloride (mmol/L)	104	104	100	102	95 - 108
Sodium bicarbonate (mmol/L)	24	23	24	28	22 - 29
Calcium (mmol/L)	2.2	-	-	2.4	2.12 – 2.60
HDL-cholesterol (mmol/L)	1.2	-	-	-	>1.0
LDL-cholesterol (mmol/L)	2	-	-	-	3.3-4.1
Total cholesterol (mmol/L)	3.3	-	-	-	<5.1
Triglyceride (mmol/L)	1.4	-	-	-	<1.7
D-dimer (g/L)	0.73	-	-	-	0.00 - 0.49
Prothrombin time/INR (s)	1.2	-	-	-	0.9 - 1.2
aPTT (s)	28	-	-	-	25 - 36
Protein C activity %	85	-	-	-	70 -140
Protein S activity %	91	-	-	-	72 - 126
Complement 3 (gm/L)	1.27	-	-	-	0.90 – 1.80
Complement 4 (gm/L)	0.30	-	-	-	0.10 – 0.40
Activated protein C resistance	1.1	-	-	-	0.91 - 1.19
Factor V Leiden variant	Negative	-	-	-	-
ANA CTD	Negative	-	-	-	-
Anticardiolipin Ab IgM	Negative	-	-	-	-
ANCA	Negative	-	-	-	-
Anti-beta 2 glycoprotein IgM	Negative	-	-	-	-
Anti-proteinase 3 antibodies	Negative	-	-	-	-
Lupus anticoagulant	Negative	-	-	-	-

**Figure 1:**
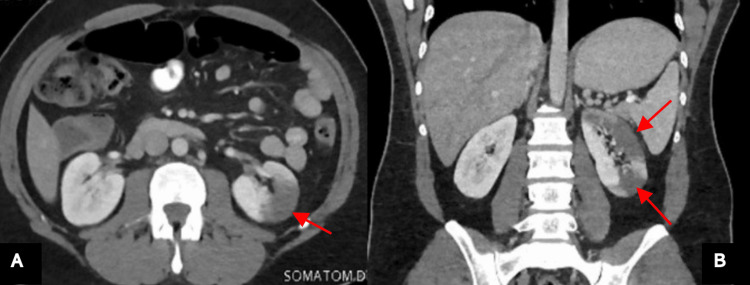
A computerized tomography scan of the abdomen and pelvis with IV contrast; the axial (A) and coronal views (B) show a wedge-shaped area of decreased enhancement in the left kidney (red arrows), consistent with renal infarction.

In the ED, lithiasic kidney disease was suspected. A computerized tomography (CT) scan of the abdomen and pelvis was obtained, which showed a wedge-shaped, non-enhanced area of the left kidney, suggestive of renal infarction (Figure 1). Given the course of the symptoms, there was no indication for interventional therapy. The chest X-ray and electrocardiogram (ECG) were normal. The patient was managed medically with therapeutic doses of enoxaparin (70 mg twice daily) and intravenous fluid. His symptoms improved, and his renal parameters showed a decreasing trend. While in the hospital, the thrombophilia and autoimmune diseases work-up was initiated and was unremarkable (Table 1). Transthoracic echocardiography (TTE) did not show any evidence of intracardiac thrombus or vegetation. Holter monitoring did not reveal any arrhythmias. The patient was discharged on day four with advice to take apixaban (5 mg) twice daily to be continued for a minimum period of six months, and further follow-up for four months was non-contributory.

## Discussion

Renal infarction is extremely uncommon, with an incidence rate of 0.1%-1.4% [4]. The actual frequency of occurrence is probably higher than what is reported because the clinical presentations are often non-specific and can mimic other common renal conditions such as urinary lithiasis or pyelonephritis [5]. The symptoms related to renal infarction include but are not limited to, flank pain, low-grade fever, nausea, vomiting, and hypertension [6]. The major etiologies of renal infarction include cardioembolic events, hypercoagulable states, and renal artery injury [7]. The mechanisms underlying thrombosis in COVID-19 are not fully understood, and several hypotheses have been implemented in the pathogenesis of this complication, which include hyperinflammatory response, direct cellular injury, cytokine storm, and complement system activation [7]. The reported prevalence of thromboembolic phenomena related to SARS-CoV-2 infection is high and considered one of the serious clinical manifestations of the disease, which may lead to catastrophic outcomes, especially in critically ill older patients [7, 8]. The complication rate of arterial and venous thrombosis increases sharply in patients with severe COVID-19 infection who require hospitalization and advanced intensive care support [8]. Interestingly, our patient, who was relatively young and had no significant risk factors for thrombosis, presented with acute thrombotic events rather than typical respiratory symptoms. We speculate that hypercoagulability may not correlate with the severity of the COVID-19 infection.

Computed tomography angiography (CTA) is useful to confirm the diagnosis and evaluation of disease extension [9]. Prompt revascularization of the renal artery should be considered, especially in patients with a solitary kidney, by assessing the kidney parenchyma threatened by the infarction, the degree of renal vessel occlusion, the estimated glomerular filtration rate (eGFR), and the duration of symptoms. The cardioembolic phenomena and hypercoagulable states should be evaluated to acknowledge the underlying etiology of renal infarction [10]. In our case, considering negative tests, the renal infarct was believed to be secondary to a hypercoagulable state from the COVID-19 infection. Angiotensin-converting enzyme (ACE) inhibitors or angiotensin receptor blockers (ARBs) should be taken into consideration to treat new-onset hypertension in patients with renal infarction. However, alternative antihypertensive agents should be considered if the patient develops acute kidney injury (AKI) or hyperkalemia [11]. Patients with renal infarction who have an identified thromboembolic risk factor should be treated with systemic anticoagulation, and concomitant antiplatelet therapy is typically administered to those who undergo stent placement or renal artery angioplasty [12].

## Conclusions

Renal infarction is a relatively uncommon condition and is underdiagnosed due to non-specific symptoms, which can mimic other common renal pathologies. The underlying mechanisms of renal infarction in COVID-19 patients are largely unknown. Thrombotic events are not correlated with the severity of COVID-19 symptoms. Physicians should include kidney infarction in the differential diagnosis of AKI in patients with COVID-19 infection for timely management and to prevent critical and irreversible kidney damage.
